# Managing dry eye disease

**Published:** 2024-10-02

**Authors:** Bruna Duarte, Monica Alves

**Affiliations:** 1 Department of Ophthalmology and Otorhinolaryngology, University of Campinas, Brazil.

**The treatment of dry eye disease aims to interrupt the vicious cycle of damage and inflammation of the ocular surface and restore the tear film.**

**Figure:**
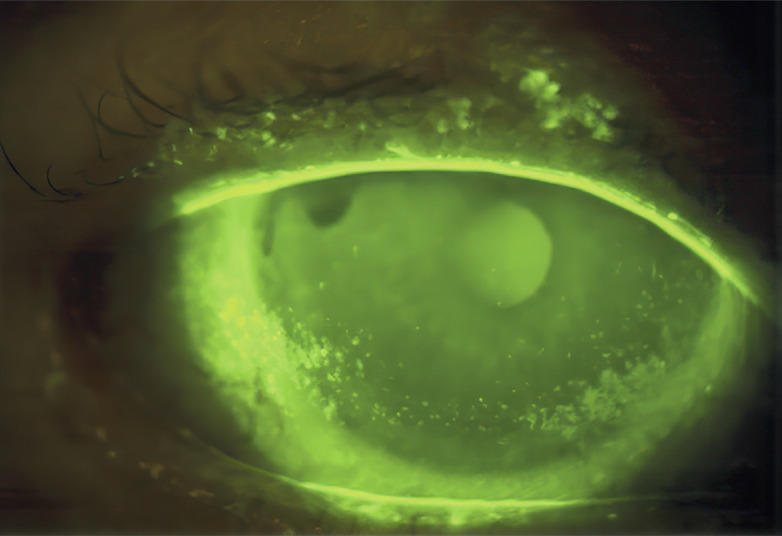
Punctate epithelial erosions characteristic of dry eye. Image taken using blue light and a yellow filter, after instilling fluorescein. UK

© VICTOR HU CC BY-NC-SA 4.0

Dry eye disease is a common, debilitating disorder of the ocular surface and one of the main reasons patients seek ophthalmic care. It affects millions of people worldwide, causing symptoms that directly impact patients’ eye health and quality of life.^1^^–^^5^ This article will concentrate on the management of dry eye disease, including recent developments.

Dry eye disease is classified based on the types of physical changes responsible (its pathophysiology). Aqueous-deficient dry eye and evaporative dry eye may overlap in variable combinations and coexist in the same clinical presentation: the mixed dry eye.^3^ In all forms, tear hyperosmolarity (when the tears are too concentrated) — whether due to reduced tear secretion, excessive evaporation, external factors, or systemic diseases — acts as a triggering factor for a cascade of events, resulting in ocular surface inflammation and damage. Tear film instability and epithelial damage stimulate and chronically damage corneal nerve endings, resulting in symptoms such as pain, increased blink rate, compensatory increase in reflex tear secretion, limitations in performing daily activities, and – often – depression.^7^

## Dry eye disease diagnosis

Dry eye disease can be distinguished from other ocular surface diseases through screening questionnaires and tests. Several diagnostic protocols have been proposed.^8^ Scores higher than 6 on the Dry Eye Questionnaire-5 (tinyurl.com/r2t35sc8) or higher than 13 on the Ocular Surface Disease Index (eyecalc.org/osdi/) may suggest dry eye disease and the need for a more detailed evaluation. The presence of reduced tear film breakup time, elevated or large differences in tear osmolarity (concentration) between eyes, or superficial ocular staining of the cornea, conjunctiva, or eyelid margin (for example, with fluorescein) are all signs of ocular surface instability that confirm the diagnosis.

After determining the presence of dry eye disease, based on positive questionnaire results and one or more positive results on eye examination, further tests — such as meibography, lipid interferometry, and tear volume measurement — may be performed (when available) to determine disease subtype and severity, and to guide management.

**Definition of dry eye**

The Dry Eye WorkShop II (DEWS II) of the Tear Film and Ocular Surface Society defined dry eye as follows (known as the DEWS II definition):

“Dry eye is a multifactorial disease of the ocular surface, characterised by a loss of homeostasis (stability) of the tear film, and accompanied by ocular symptoms, in which tear film instability and hyperosmolarity (concentration), ocular surface inflammation and damage, and neurosensory abnormalities play etiological (causative) roles.”^6^

## Dry eye disease management

Dry eye disease management is challenging as the causes are often complex. Treatment aims to interrupt the vicious cycle of damage and inflammation by restoring tear film and ocular surface stability. In this article, we propose a 4-step approach to the management of dry eye disease.

### Step 1. Laying a good foundation

The strategies in this step are the foundation of dry eye disease management. They must be continued alongside any other treatment the patient may receive in future.

#### Education & counselling

Start by educating the patient about the disease, including what can make it worse, and how to manage it at home. Encourage patients to reduce the time spent looking at computer or phone screens and/or to take breaks when using screens. Taking regular exercise, and avoiding air conditioning, smoke, or other pollutants, can also help.

#### Tear supplementation

Prescribing preservative-free artificial tears is a common strategy.

- **Aqueous eye drops** can contain agents such as hyaluronic acid and hydroxypropyl guar; these make the eye drops more viscous (thicker), which keeps them on the ocular surface for longer.
- **Lipid-based eye drops** and **liposomal sprays** have become more popular for the management of signs and symptoms associated with meibomian gland dysfunction and lacrimal lipid deficiency.^1^

All formulations should be preservative free if at all possible as preservatives in eye drops can cause toxicity and worsening of the dry eye disease.

#### Eyelid care

Eyelid hygiene practices such as warm compresses (followed by eyelid massage), and eyelid cleaning (e.g., using cooled boiled water and a clean face cloth or cotton wool pad) help alleviate blepharitis and meibomian gland dysfunction symptoms, thereby promoting tear film stability. Such strategies should be used from the beginning and often help improve dry eye disease when performed properly. See the previous article in this issue for more information.

#### Dietary modifications

Encourage patients to drink plenty of water and eat more foods that contain essential fatty acids (EFAs) such as oily fish, nuts, seeds, and green, leafy vegetables. EFAs, particularly omega-3 and omega-6, play a significant role in modulating inflammatory mechanisms in the human body. While laboratory studies demonstrate the anti-inflammatory properties of omega-3 and its positive effect on lacrimal gland function and the ocular surface, clinical trials investigating oral EFA supplementation for dry eye disease treatment have shown varied results; there are also controversies surrounding the ideal dosage, formulation, and duration of treatment. Nevertheless, omega-3 supplementation is widely used and is part of the authors' list of initial therapies proposed for the treatment of dry eye disease.

#### Environmental and behavioural control measures

Certain behavioural and environmental conditions can also play a role in the development and progression of dry eye disease. Exposure to dry or polluted air can aggravate dry eye symptoms, as can excessive screen exposure time and sedentary habits. Therefore, it is important to advise patients about their habits and environment and seek ways to reduce or compensate for harmful factors, such as scheduled breaks during screen use, environmental adjustments (e.g., the use of humidifiers in very dry environments), taking regular physical exercise, and avoiding air conditioning, smoke, or other pollutants.

#### Review of medications

The use of systemic medications for other purposes can affect the ocular surface. For instance, antihistamines, antidepressants, anxiolytics, and isotretinoin (for acne) are known for their adverse effects on the ocular surface. Depending on the severity and significance of the adverse effects, both physician and patient may choose to reduce, replace, or discontinue the medication. Long term use of glaucoma drops is often associated with dry eye disease, especially prostaglandin analogue drops. Any eye drops being used should also be reviewed and reduced or changed to preservative-free formulations if possible.

### Step 2. Stepping up treatment

In patients where the measures in Step 1 prove ineffective or insufficient, there is a need to step up treatment. In this stage, it is possible to increase lubricating measures, evaluate the possibility of *Demodex* infestation, and consider the use of tear conservation devices, meibomian gland dysfunction treatment therapies, and other specific topical prescriptions.

#### Tear supplementation

Increase lubricating measures by prescribing aqueous or lipid-based gels and ointments.

#### Treatment for *Demodex*

The infestation of the eyelid margins by the *Demodex folliculorum* mite might be considered in patients with resistant blepharitis. This a common condition that can cause symptoms such as itching, redness, and irritation. Management often includes **topical tea tree oil-based solutions or ointments** and **eyelid hygiene** practices (see Step 1), as well as oral medication containing **ivermectin**.

#### Tear conservation

Interventions that aim to promote tear conservation can also be valuable in this stage of treatment. **Temporary punctal occlusion** with punctal plugs involves closure of the proximal tear drainage ducts in order to retain natural tears and prolong contact with the ocular surface. **Moisture chamber spectacles or goggles** can be used to create a barrier and help reduce tear evaporation; they can also promote comfort and improve patients’ quality of life during treatment.

#### Meibomian gland dysfunction treatment

In patients for whom the clinical approach in Step 1 is not enough, there may be a need for further interventions, although access to these may be limited. **Intense pulsed light** (IPL) can be used to target dysfunctional blood vessels and decrease inflammation in the eyelids and surrounding tissue, thereby improving tear film stability. **Thermal pulsation therapy**, often followed by **mechanical gland expression**, helps address meibomian gland dysfunction by applying controlled heat to the eyelids; this helps the release of meibum. Eyelid treatments such as debridement and exfoliation aim to remove debris and biofilm from the eyelid margins, promoting healthier tear production and relieving symptoms.^5^ These strategies might be used alone or in combination, depending on the patient's needs.

#### Other drug options

Other options can be added, e.g. if there are specific signs, progression or acute worsening of symptoms, or if there is no response to previously described treatment.

- **Topical corticosteroids** play a crucial role in managing various inflammatory conditions, including dry eye disease, by inhibiting the inflammatory cascade. It must be administered for a limited duration and in a tapering regimen, as long-term use is associated with potential complications such as cataract, ocular hypertension, and opportunistic infections.
- **Non-steroidal drugs that affect the immune system** (immunomodulators) offer a promising alternative to corticosteroids. Cyclosporine A, the most studied immunomodulator so far, has been approved for moderate to severe dry eye disease as it showed significant reduction in inflammatory markers and improvement in tear osmolarity.^4^ Tacrolimus, a substance with superior immunosuppressive potential, has emerged as a viable therapeutic option for patients that are intolerant of, or unresponsive to, cyclosporine A. However, tacrolimus is not widely available.^1^
- A wide range of **topical formulations for lacrimal secretion stimulation** (known as secretagogues) are known but not widely available. These vary in mechanism of action, stimulating aqueous, mucous, and/or lipid secretion. Notable topical secretagogues include diquafosol tetrasodium and lacritin, which stimulate aqueous-mucous secretion and promote lacrimal gland secretory processes, respectively.
- **Topical mucolytics** are often used as adjunctive therapy, especially those with antioxidant properties, such as acetylcysteine. These work by breaking down mucous (or mucin) molecules. These medications may also be used to treat filamentary keratitis, a potential complication of dry eye.
- Finally, **systemic and topical antibiotics**, such as tetracyclines and azithromycin, are prescribed not only for their antimicrobial effects but also for their anti-inflammatory properties in conditions associated with dry eye disease, such as blepharitis and meibomian gland dysfunction.^5^ Optimal dosages and protocols for these antibiotics remain controversial, and further research is needed to determine their efficacy and long-term effects.^1^ Examples of commonly used regimens include doxycyline 50–100 mg, or lymecycline 408 mg, used once a day for several months. Note their contraindications, including use in pregnancy and young children.

### Step 3. Long-term management

In this step, there is an even greater concern about the long-term effects and sequelae of the disease. Further approaches have to be considered and potentially added to the overall treatment in very severe cases.

#### Oral secretagogues

Oral secretagogues can be used to help increase tear secretion, although their effective role remains controversial. **Pilocarpine** and **cevimeline** are cholinergic agonists that can be administered orally to manage especially moderate to severe dry eye disease.^1^

#### Biological tear substitutes

Biological tear substitutes have also been extensively used in treating severe ocular surface diseases due to their biochemical similarities to tears. Examples include **autologous and allogeneic serums**, derived from the blood of the patient or from another human being, respectively. While complications are rare, concerns over contamination, lack of manufacturing standardisation, and immune response risks tend to limit its widespread use. The use of **platelet-rich plasma** (PRP) is also a valuable therapeutic option due to its superior growth factor content compared to fresh frozen plasma or serum. However, there is also a lack of consensus on manufacturing protocols, which means further research is needed to fully assess their benefits and indications in dry eye disease treatment.^1^

#### Therapeutic contact lens options

Therapeutic contact lenses can be helpful, providing symptomatic relief and improving ocular comfort for patients with moderate to severe dry eye disease. **Soft bandage lenses** provide a soothing barrier over the cornea, retaining tears for a longer period and playing a role in the healing of epithelial lesions. On the other hand, **rigid scleral lenses** can create a vault over the corneal extension and provide a tear reservoir between the lens and the epithelium, thereby hydrating and protecting the ocular surface.

### Step 4. More permanent solutions

The final step of treatment for severe dry eye disease consists of long-term topical and/or oral medications, as well as more invasive approaches such as surgical procedures involving permanent punctal occlusion, amniotic membrane graft, tarsorrhaphy, and salivary gland transplantation. Fortunately, only a small number of patients tend to present with such severe forms of dry eye disease.

#### Prescription drug options

After all the clinical attempts mentioned in the previous steps, the use of long-term **topical/oral corticosteroids and oral immunosuppressants** need to be considered.

#### Surgical approaches

Surgical alternatives for dry eye disease include various approaches to alleviate symptoms and improve ocular surface health. Correction of eyelid malposition with **ectropion or entropion surger**y should be considered if appropriate. Permanent occlusion of the lacrimal puncta with **punctal cautery** can be considered if there is significant aqueous deficiency, e.g., in Sjögren's syndrome, and if a trial of punctal plugs does not cause watering of the eyes (initially on the lower puncti only). **Tarsorrhaphy** to reduce (partially or completely) the exposure of the ocular surface and to decrease tear film evaporation would normally be reserved for patients with serious, vision-threatening disease. Surgical procedures utilising **amniotic membrane grafts** can restore tissue integrity and reduce inflammation, but these are temporary measures and are normally only considered in very severe disease. **Major salivary gland transplantation** and **autotransplantation of minor salivary glands** from oral and nasal mucosa can also aid in reconstructing the fornices and in managing dry eye disease symptoms; however, this requires a very specialised service to be available.^1^

Regular monitoring and careful long-term evaluation are crucial for ensuring the effectiveness of the proposed therapies, making it possible to modify or complement them as needed. Additionally, patient education and participation in decision-making have a significant impact on treatment success and adherence, as well as assisting in the prevention of relapses and complications.

## Summary

This article represents a synthesis of the current knowledge about dry eye disease, with a focus on management. It is important to understand the multifaceted nature of this condition and the variety of therapeutic approaches available.

There are numerous opportunities for further research and many fields of innovation and investigation are yet to be explored. Commitment to clinical excellence and continuous medical education are essential if we want to promote the wellbeing and quality of life for our dry eye disease patients.
